# The Distribution and Diversity of *Bartonella* Species in Rodents and Their Ectoparasites across Thailand

**DOI:** 10.1371/journal.pone.0140856

**Published:** 2015-10-20

**Authors:** Kewalin Klangthong, Sommai Promsthaporn, Surachai Leepitakrat, Anthony L. Schuster, Patrick W. McCardle, Michael Kosoy, Ratree Takhampunya

**Affiliations:** 1 Department of Entomology, United States Army Medical Directorate—Armed Forces Research Institute of Medical Sciences, Bangkok, Thailand; 2 Division of Vector-Borne Diseases, Centers for Disease Control and Prevention, Fort Collins, Colorado, United States of America; University of the Sunshine Coast, AUSTRALIA

## Abstract

Our study highlights the surveillance of *Bartonella* species among rodents and their associated ectoparasites (ticks, fleas, lice, and mites) in several regions across Thailand. A total of 619 rodents and 554 pooled ectoparasites (287 mite pools, 62 flea pools, 35 louse pools, and 170 tick pools) were collected from 8 provinces within 4 regions of Thailand. *Bandicota indica* (279), *Rattus rattus* (163), and *R*. *exulans* (96) were the most prevalent species of rats collected in this study. Real-time PCR assay targeting *Bartonella*-specific *ssr*A gene was used for screening and each positive sample was confirmed by PCR using *nuo*G gene. The prevalence of *Bartonella* DNA in rodent (around 17%) was recorded in all regions. The highest prevalence of *Bartonella* species was found in *B*. *savilei* and *R*. *rattus* with the rate of 35.7% (5/14) and 32.5% (53/163), respectively. High prevalence of *Bartonella*-positive rodent was also found in *B*. *indica* (15.1%, 42/279), and *R*. *norvegicus* (12.5%, 5/40). In contrast, the prevalence of *Bartonella* species in ectoparasites collected from the rats varied significantly according to types of ectoparasites. A high prevalence of *Bartonella* DNA was found in louse pools (*Polyplax* spp. and *Hoplopleura* spp., 57.1%) and flea pools (*Xenopsylla cheopis*, 25.8%), while a low prevalence was found in pools of mites (*Leptotrombidium* spp. and *Ascoschoengastia* spp., 1.7%) and ticks (*Haemaphysalis* spp., 3.5%). Prevalence of *Bartonella* DNA in ectoparasites collected from *Bartonella*-positive rodents (19.4%) was significantly higher comparing to ectoparasites from *Bartonella*-negative rodents (8.7%). The phylogenetic analysis of 41 *glt*A sequences of 16 *Bartonella* isolates from rodent blood and 25 *Bartonella*-positive ectoparasites revealed a wide range of diversity among *Bartonella* species with a majority of sequences (61.0%) belonging to *Bartonella elizabethae* complex (11 rodents, 1 mite pool, and 5 louse pools), while the remaining sequences were identical to *B*. *phoceensis* (17.1%, 1 mite pool, 5 louse pools, and 1 tick pool), *B*. *coopersplainensis* (19.5%, 5 rodents, 1 louse pool, and 2 tick pools), and one previously unidentified *Bartonella* species (2.4%, 1 louse pool).

## Introduction

*Bartonella* bacteria are new emerging pathogens causing diseases in humans and animals [1, 2]. The members of genus *Bartonella* are rod-shaped gram negative facultative intracellular bacteria that are fastidious and slow growing at aerobic conditions. They infect human and other mammalian hosts via infected-vectors such as fleas, ticks, and lice or the bite/scratch of an infected-animal [3–5]. Moreover, the infected arthropods could transmit *Bartonella* bacteria to human and other mammalian hosts via feces through superficial scratches in skin [6]; for example, *B*. *henselae* and *B*. *quintana* were transmitted to hosts via contaminated feces of infected cat fleas (*Ctenocephalides felis*) and human body lice (*Pediculus humanus*), respectively [7]. Pathogenesis involves the invasion of host’s erythrocytes, endothelial cells, and dendritic cells which play an important role in the first line immune response to fight against pathogens [8, 9]. As a result of the immune system failure, a bacteremia persistent infection might occur [8, 10].

*Bartonella* genus comprises over 30 species and subspecies [11]. At least thirteen known or suspected species are thought to contribute to blood-borne infections in human [12]. Moreover, several studies suggested the role of *Bartonella* species as a potential causative agent for cases of unknown febrile illness as well as endocarditis in patients in Thailand [13]. The diversity of *Bartonella* species in several countries in Southeast Asia (Lao PDR, Cambodia, and Thailand) has been reported and the findings revealed that *Bartonella* species in rodents are much more diverse than in other animals, except bats. The species found in rodents included *B*. *elizabethae*, *B*. *coopersplainsensis*, *B*. *phoceensis*, *B*. *queenslandensis*, *B*. *rattimassiliensis*, *B*. *tribocorum* and three genotypes presumably representing new *Bartonella* species [14].

*Bartonella* transmission occurs mainly via horizontal transmission when arthropod vectors acquire *Bartonella* bacteria during the feeding of infected host and later they become infected and the infected vectors then transfer the bacteria to another host [5, 15]. Interestingly, some studies suggested that vertical and transstadial transmissions of *Bartonella* species in *Ixodes* ticks [16], deer ked [17], and transplacental in rodent populations [18].

High prevalence of *Bartonella* DNA and genotype diversity have been detected in arthropod vectors around the world. For example, ticks collected from dogs and donkeys in Peru were found to carry several *Bartonella* species, such as *B*. *rochalimae*, *B*. *quintana* and *B*. *elizabethae* [19]. In Taiwan, *B*. *tribocorum*, *B*. *elizabethae*, *B*. *queenslandensis*, *B*. *rochalimae*-like bacteria, *B*. *phoceensis*, and *B*. *rattimassiliensis* were detected in fleas and louse pools [20]. Several studies in Thailand have reported the detection of *B*. *henselae*, *B*. *clarridgeiae*, and *B*. *koehlerae* from cats and flea pools collected from the Thai-Myanmar border [21] and in the Bangkok area [22]. Moreover, novel species such as *B*. *tamiae* was recently isolated from whole blood of febrile patients from Thailand [23] and DNA belonging to this species was also detected from the pools of ticks and mites collected from rats in Thailand [15].

Though a number of papers on *Bartonella* in rodents from Thailand have been published, the comparative analysis of bartonellae between rodent hosts and ectoparasites has not been done. Our aim was to investigate the prevalence and diversity of *Bartonella* species in rodents and their ectoparasites, and to estimate the importance of this host-vector relationship for the transmission of *Bartonella* species in natural habitats of Thailand. Our results indicated a significant difference between bacterial communities recognized in mammals and arthropods.

## Materials and Methods

### Study sites and samples processing

The study sites were located in different regions of Thailand. Rodents and their associated ectoparasites (ticks, fleas, mites, and lice) were collected from eight provinces within four regions of Thailand during the period of December 2012 to November 2013. The regions included the Northern region (Chiang Rai and Phayao provinces), the Southern region (Chumphon and Surat Thani provinces), the Eastern region (Rayong and Trat provinces) and the Northeastern region (Loei and Nong Bua Lam Phu provinces) (Table 1). This study was carried out on private lands and the owners of the lands gave permission to conduct the study on their sites and the field studies did not involve endangered or protected species. Rodents were captured by live traps baited with bananas or dried fish. Rodents were collected from orchards, cultivated rice-fields, grassland areas, edges of dense forest, stream margins, and around houses. Traps were set for 3–5 nights and were checked early in the morning. Then, rodents were removed from the traps and later identified to species [24]. Captured rodents were killed by carbon dioxide and processed on the same day and at the site of capture. Blood and serum samples and rodent tissue samples (liver, spleen, kidney and lung) were collected and stored on dry ice, and transported to the AFRIMS laboratory. Rodent’s ears were cut and stored in 70% ethanol for mite collections and the other ectoparasites (ticks, fleas, and lice) were collected from individual rodents by combing and stored in 70% ethanol for transportation to the laboratory. Mites in their larval stage (chigger) were collected from rodent’s ears by paintbrush under the stereomicroscope and pooled by host. Three to five mites were selected from each pool and mounted on glass slides for morphological identification to genera and species if possible using taxonomic key [25]. Ectoparasites of each type (fleas, ticks, and lice) were identified morphologically [26, 27] and pooled by host, type, stage, and gender in 1.5 ml microcentrifuge tube. Pools of ectoparasites were subjected to DNA extraction procedures as described below. Louse species identification was performed following the previously published protocol [28]. Details of the ectoparasites collected from rodents in this study are provided in Table A in S1 File.

**Table 1:** Location coordinates of rodents and ectoparasites collection sites in Thailand (2012–2013).

Regions	Provinces	Districts	Sub-districts	Villages	Latitude	Longitude
**North**	**Chiangrai**	Mae Chan	Chanchawatai	Ban Pagook	20°15' 16.042'' N	99°56' 2.144'' E
		Mae Chan	Pa Sang	Rong Khi	20°10' 47.543'' N	99°50' 48.505'' E
	**Phayao**	Dok Khamtai	Ban Tham	Ban Sansai	19°6' 54.234'' N	100°3' 44.319'' E
** **	** **	Dok Khamtai	Ban Tham	Ban Tham Mongkol	19°6' 2.606'' N	100°2' 25.71'' E
**Northeast**	**Loei**	Dan Sai	Na Di	Ban Na Mue Muen	17°19' 15.859'' N	101°8' 58.304'' E
		Dan Sai	Na Di	Ban Na Ho	17°19' 30.922'' N	101°8' 51.766'' E
		Dan Sai	Pak Man	Ban Pak Man	17°29' 42.137'' N	101°10' 48.572'' E
	**NongBua Lam Phu**	Si Bun Rueang	Non Sa-at	Ban Wang Khaen	16°55' 9.883'' N	102°8' 58.138'' E
** **	** **	Si Bun Rueang	Na Kok	Ban Non Ngam	16°53' 42.23'' N	102°13' 5.375'' E
**East**	**Rayong**	Pluak Daeng	Map Yang Phon	Sapan Seeyakmahanakhorn	13°0' 59.404'' N	101°8' 16.778'' E
		Pluak Daeng	Map Yang Phon	Sasithorn16	13°0' 48.629'' N	101°7' 5.664'' E
		Pluak Daeng	Map Yang Phon	Bo Win	13°1' 23.012'' N	101°6' 38.523'' E
	**Trat**	Khlong Yai	Khlong Yai	Ban Suan Maprao	11°46' 51.946'' N	102°52' 36.364'' E
** **	** **	Khlong Yai	Hat Lek	Ban Khlong Son	11°43' 52.561'' N	102°54' 1.699'' E
**South**	**Chumphon**	Tha Sae	Tha Kham	Ban Dinkong	10°39' 34.463'' N	99°6' 11.62'' E
		Mueang	Bangluek	Ban Salaloy	10°39' 34.650'' N	99°6' 12.592'' E
		Mueang	Bangluek	Ban Nongnean	10°34' 23.675'' N	99°12' 56.937'' E
	**Surat Thani**	Mueang	Wat Pradu	Wat Ma Pring	9°6' 59.065'' N	99°16' 34.42'' E
	** **	Phunphin	Khao Hua Khwai	Bang Or	9°5' 16.771'' N	99°13' 14.005'' E

Surveillance activities were conducted in 4 regions and 8 provinces in Thailand. The Northern region (Chiangrai and Phayao provinces), the Northeastern region (Loei and Nong Bua Lam Phu provinces), and the Southern region (Chumphon and Surat Thani provinces).

### Genomic DNA extraction from rodent tissue and ectoparasites

Genomic DNA was extracted from rodent livers using the Wizard® Genomic DNA purification kit (Promega, Madison, WI) according to the manufacturer’s instructions with some modifications. Briefly, the liver tissue was cut into pieces of approximately 3 millimeters in diameter and added to 600 μl of Nuclei Lysis Solution (Promega, Madison, WI). The mixture was homogenized with beads using a TissueLyser II machine (Qiagen, Hilden, Germany) at 25 Hz for 5 min twice. Subsequently, the mixture was incubated with 20 μl of Proteinase K solution (20 mg/ml) at 55°C for 1 hr, and then with 3 μl of RNase A (10 mg/ml) at 37°C for 15 min. Then 200 μl of protein precipitation solution (Promega, Madison, WI) was added and mixed vigorously by vortex. The mixture was kept on ice for 5 min. Insoluble materials were removed by centrifugation at 13,000 rpm for 4 min and the supernatant was transferred to a new tube. DNA was precipitated by adding 600 μl of isopropanol and then centrifuged at 13,000 rpm for 1 min. DNA pellet was washed using 70% ethanol and dried by SpeedVac™ concentrator (Thermo Scientific, Waltham, MA). Two hundred microliters of EB buffer (10 mM Tris Cl, pH 8.5) were used to resuspend dried DNA and stored at -20°C until further analysis.

DNA extraction from the ectoparasites was performed according to the tissue extraction protocol from QIAamp^®^ DNA Mini Kit (Qiagen, Hilden, Germany) with some modification. Briefly, pools of ticks, fleas and lice were puncture in the presence of liquid nitrogen in a 1.5 ml microcentrifuge tube. Mites were punctured with a fine needle under microscopy. Next, ninety microliters of ATL lysis buffer were added to each sample and mixed thoroughly. Then, ten microliters of Proteinase K solution (20 mg/ml) was added and incubated at 56°C for 3 hr. One hundred microliters of AL buffer was added to the samples and mixed by pulse-vortexing for 15 sec then incubated at 70°C for 10 min. After that, 100 μl of absolute ethanol was added and the mixture was mixed by pulse-vortexing for 15 sec. Finally, the mixture was transferred to a QIAamp spin column and DNA was eluted in 50 l AE buffer. DNA solution was stored at -20°C until further analysis.

### *Bartonella* detection in rodent tissues and ectoparasites

DNA extracts obtained from rodent tissues and ectoparasites were screened for the presence of *Bartonella* species using real-time PCR assay (qPCR) with TaqMan probe. A genus-specific assay targeting a transfer-mRNA gene (*ssr*A) of *Bartonella* species was used in this study following previously published protocol [29]. The primer pair, *ssr*A-F/ *ssr*A-R, and *ssr*A-probe were used to amplify 301 bp fragment of *ssr*A gene. The qPCR reaction (25 μl) consisted of 12.5 μl Platinum® Quantitative PCR SuperMix-UDG (Invitrogen, Grand Island, NY), 0.5 μM of each primer, and 0.1 μM of *ssr*A-Probe and 2 μl of DNA template or nuclease free water as non-template control. The qPCR conditions were performed as follows: UDG incubation at 50°C for 2 min and then initial denaturation at 95°C for 2 min followed by 45 cycles of 95°C for 15 sec and 60°C for 30 sec using the Chromo4™ Real-Time Detector (Bio-Rad, Hercules, CA). Every sample with a positive signal from the screening assay was subjected to confirmatory test. A different target gene of NADH Dehydrogenase Gamma Subunit gene (*nuo*G) of *Bartonella* species was used to confirm the positivity by conventional PCR assay. A 346 bp fragment of *nuo*G gene was amplified with *nuo*G-F and *nuo*G-R primer pair according to previously published protocol [30]. The PCR reaction mixture (25 μl) consisted of 1X of PCR buffer, 0.2 μM of each primer, 0.2 mM of dNTP, 1.25 U of Taq DNA Polymerase (Invitrogen, Grand Island, NY) and 5 μl of DNA template or nuclease free water as non-template control. PCR amplification was carried out using the Veriti® 96-well Thermal Cycler (Applied Biosystems, Foster City, CA) with the initial denaturation at 94°C for 3 min followed by 45 cycles of denaturation at 94°C for 45 sec, annealing at 55°C for 1 min and extension at 72°C for 1 min 30 sec, then incubated at 72°C for 10 min for the final extension step. The amplification product of 346 bp was observed with 1.5% agarose gel electrophoresis under the UV visualization.

### *Bartonella* culture from rodent blood

Selected rodents, which were *Bartonella*-positive by molecular assays, were subjected to *Bartonella* culture following previously published protocol [31]. At the field sample site, whole blood was collected from each rodent by cardiac puncture and preserved in EDTA. Rodent whole blood was kept in -70°C until use. Briefly, whole blood was retrieved from the -70°C freezer and thawed at 4°C. Then, homogeneous whole blood was diluted 1:4 in 1X Dulbecco’s Phosphate Buffered Saline (GIBCO, Grand Island, NY) containing 5–10% Fungizone (GIBCO, Grand Island, NY). Diluted blood sample (0.1 ml) was pipetted onto Brain Heart Infusion agar plates containing 5% rabbit blood (BBL, Becton Dickinson Microbiology Systems, Cockeysville, MD). Four to five agar plates were kept in a plastic bag and incubated at 37°C, 5% CO_2_ for up to 4 weeks. The agar plates were monitored once a week after the initial inoculation and once per three days after sub-culturing. Sub-culturing was continued until a pure culture was obtained. *Bartonella*-like colonies were recognized by colony morphology and then harvested into 10% sterile glycerol and kept in -70°C freezer for further confirmation and characterization. A portion of each *Bartonella*-like colony was subjected to DNA extraction using QIAamp DNA Mini Kit (Qiagen, Hilden, Germany) following manufacturer’s instruction and confirmed to be *Bartonella* species by citrate synthase gene (*glt*A) sequence identity.

### Citrate synthase gene (*glt*A) amplification

Amplification of *Bartonella glt*A gene was done using published primers, BhCS.781p and BhCS.1137n [32]. The PCR reaction (50 μL) consisted of 1X of PCR buffer, 2.5 mM of MgCl_2_, 0.2 μM of each primer, 0.2 mM of dNTP, 2.5 U of AmpliTaq Gold® DNA Polymerase (Applied Biosystems, Foster City, CA) and 2.5 μl of DNA template or nuclease free water as non-template control. The amplification conditions were as follows: the initial denaturation at 95°C for 3 min followed by 35 cycles of denaturation at 95°C for 1 min, annealing at 56°C for 1 min and extension at 72°C for 1 min, then the final extension step at 72°C for 10 min. The amplification product (379 bp) was observed on 1.5% agarose gel electrophoresis.

### DNA sequencing

Amplification products of *ssr*A or *glt*A genes were purified using QIAquick PCR Purification Kit (QIAGEN Inc., Valencia, CA) following the manufacturer’s instruction and sent for sequencing at AITbiotech Pte. Ltd. (Singapore)

### Statistical analyses

The difference in the prevalence of *Bartonella* DNA in ectoparasites collected from *Bartonella*-positive and *Bartonella*-negative rodents was confirmed by Chi-Square Test and the critical range (*P* < 0.005) was used. The statistical calculations were performed with IBM® SPSS® Statisic (version 22) software (Chicago, IL).

### Sequence and Phylogenetic analysis

Sequence data were assembled using the Sequencher 5.1 software (Gene Code Corporation, Ann Arbor, MI) and the consensus sequences were used for analyses. Sequences were aligned and constructed a similarity matrix with the reference sequences of *Bartonella* species using Muscle algorithm implemented in MEGA 6.0 software [33]. Maximum likelihood (ML) trees based on Kimura’s 2-parameter model (K2+G+I) were constructed using molecular evolutionary genetics analysis (MEGA) 6.0 software [33] and bootstrap analyses with 1,000 resamplings performed to test the robustness of the branching.

### Ethics Statement

Rodents trapping were carried out in the different locations of the provinces according to the institutional animal collection protocol entitled “Field Sampling of Small Mammal (Orders: Erinaceomorpha; Soricomorpha; Scandentia; Macroscelidea and Rodentia) Populations to Support Zoonotic Diseases Surveillance and Ectoparasite Collection” (PN# 12–06) reviewed and approved by the USAMC-AFRIMS Institutional Animal Care and Use Committee (IACUC). All sampling procedures and experimental manipulations were reviewed and approved as part of obtaining the animal collection protocol (PN# 12–06). Research was conducted in compliance with the Animal Welfare Act and other federal statutes and regulations relating to animals and experiments involving animals and adheres to principles stated in the Guide for the Care and Use of Laboratory Animals, NRC Publication, 2011 edition.

## Results

### Prevalence rate of *Bartonella* species among rodent and their associated ectoparasites in Thailand

A total of 619 rodents, 287 mite pools (3–153 mites/pool), 62 flea pools (1–14 fleas/pool), 35 louse pools (1–20 lice/pool), and 170 tick pools (1–24 ticks/pool) were collected from eight provinces within four regions of Thailand. Overall prevalence of *Bartonella* species in rodents was 17.6% (109/619); and prevalence in their associated ectoparasites varied substantially depending on ectoparasite type (Table 2). The highest prevalence was found in lice (57.1%, 20/35 pools) followed by fleas [25.8%, 16/62 pools (15 pools of females and 1 pool of males, Table B in S1 File)], with evident decline in ticks (3.5%, 6/170 pools of female ticks, Table C in S1 File) and mites (1.7%, 5/287 pools). A high *Bartonella* prevalence was found in rats collected from the North (22.9% in Phayao), the East (25.6% in Rayong), and the South (26.6% in Chumphon and 28.3% in Surat Thani), and a high prevalence in their ectoparasites was found in Eastern regions (23.3% in Rayong and 30.8% in Trat) as shown in Table 2.

**Table 2:** Prevalence of *Bartonella* DNA among wild-caught rodents and their associated ectoparasites collected from different regions and provinces in Thailand, 2012–2013.

		No. of *Bartonella* DNA positive/total collected (% positive)^a^					
			Rodent-associated ectoparasites				
Region	Provinces	Rodents	Mite pool	Flea pool	Louse pool	Tick pool	Total ectoparasites
**North**	Chiangrai	19/138 (13.8)	0/57 (0)	-	1/2 (50.0)	-	**1/59 (1.7)**
**North**	Phayao	14/61 (22.9)	0/15 (0)	0/2 (0)	-	5/157 (3.2)^#^	**5/174 (2.9)**
**Northeast**	Loei	6/53 (11.3)	0/25 (0)	3/11 (27.3)	-	0/7 (0)	**3/43 (7.0)**
**Northeast**	NongBua Lam Phu	3/42 (7.1)	0/8 (0)	2/26 (7.7)^£^	0/1 (0)	-	**2/35 (5.7)**
**East**	Rayong	10/39 (25.6)	0/14 (0)	1/2 (50.0)	5/8 (62.5)	1/6 (16.7)	**7/30 (23.3)**
**East**	Trat	6/101 (5.9)	0/15 (0)	9/20 (45.0)	3/4 (75.0)	-	**12/39 (30.8)**
**South**	Chumphon	21/79 (26.6)	5/73 (6.8)	-	9/16 (56.3)	-	**14/89 (15.7)**
**South**	Surat Thani	30/106 (28.3)	0/80 (0)	1/1 (100.0)	2/4 (50.0)	-	**3/85 (3.5)**
	**Total**	**109/619 (17.6)**	**5/287 (1.7)**	**16/62 (25.8)**	**20/35 (57.1)**	**6/170 (3.5)**	**47/554 (8.5)**

^a^ *Bartonella* DNA was detected by *ssr*A and *nuo*G genes. The positivity for each sample was recorded only when 2 assays produced the concordant results.

^#^ Two *B*. *indica* rats had 2 positive tick pools.

^£^ One *R*. *exulans* had 2 positive flea pools.

### Prevalence of *Bartonella* DNA in rodent species and their associated ectoparasites (mite, louse, flea, and tick)

*Bandicota indica* (45.1%, 279/619), *Rattus rattus* (26.3%, 163/619) and *R*. *exulans* (15.8%, 98/619) were the rodent species with highest rates of *Bartonella* prevalence (Table 3). A high prevalence of *Bartonella* DNA was also found in *B*. *savilei* (35.7%) and *R*. *rattus* (32.5%) followed by *R*. *sabanus* (16.7%), *B*. *indica* (15.1%), *R*. *norvegicus* (12.5%), and *R*. *exulans* (3.1%). Rats of *B*. *indica* and *R*. *rattus* were heavily infested with a variety of ectoparasites (mite, tick, and louse) accounting for 84.3% of all ectoparasites collected from rodents in this study (Table 4).

**Table 3:** Distribution of *Bartonella* DNA among rodent species in different regions and provinces of Thailand, 2012–2013.

	No. of *Bartonella* DNA positive/total collected (% positive)^a^								
	North		Northeast		East		South		
Rodent species	Chiangrai	Phayao	Loei	Nong Bua Lam Phu	Rayong	Trat	Chumphon	Surat Thani	Total
***B*. *indica***	17/119(14.3)	11/56(19.6)	1/4(25.0)	1/1(100)	0/2(0)	0/13(0)	0/20(0)	12/64(18.8)	**42/279(15.1)**
***B*. *savilei***	2/9(22.2)	-	-	2/4(50.0)	-	-	1/1(100)	-	**5/14(35.7)**
***Mus caroli***	-	-	0/1(0)	-	-	-	-	-	**0/1(0)**
***M*. *cervicolor***	0/1(0)	-	-	-	0/2(0)	-	-	-	**0/3(0)**
***R*. *berdmorei***	-	-	-	-	-	-	0/1(0)	-	**0/1(0)**
***R*. *bukit***	-	-	0/3(0)	-	-	-	-	-	**0/3(0)**
***R*.*exulans***	0/3(0)	2/2(100)	1/26 (3.8)	0/33(0)	0/1(0)	0/28(0)	0/1(0)	0/2(0)	**3/96(3.1)**
***R*. *losea***	0/3(0)	-	-	-	-	-	-	-	**0/3(0)**
***R*. *norvegicus***	-	-	-	-	3/17(17.6)	2/23(8.7)	-	-	**5/40(12.5)**
***R*. *rattus***	0/3(0)	1/3(33.3)	3/3(100)	0/4(0)	7/17(41.2)	4/37(10.8)	20/56(35.7)	18/40(45.0)	**53/163(32.5)**
***R*. *sabanus***	-	-	1/6(16.7)	-	-	-	-	-	**1/6(16.7)**
***R*. *surifer***	-	-	0/10(0)	-	-	-	-	-	**0/10(0)**
**Total**	**19/138(13.8)**	**14/61(22.9)**	**6/53(11.3)**	**3/42(7.1)**	**10/39(25.6)**	**6/101(5.9)**	**21/79(26.6)**	**30/106(28.3)**	**109/619(17.6)**

^a^ *Bartonella* DNA was detected by *ssr*A and *nuo*G genes. The positivity for each sample was recorded only when 2 assays produced the concordant results.

**Table 4:** Prevalence of *Bartonella* DNA-positive ectoparasites by rodent species based on detection of the *ssr*A gene fragment.

	No. of *Bartonella* DNA-positive pools/total collected (% positive)				
Host species	Mites	Fleas	Lice	Ticks	All ectoparasites
***B*. *indica***	0/153 (0)	0/5 (0)	1/3 (33.3)	5/153 (3.3)	6/314 (1.9)
***B*. *savilei***	0/9 (0)	-	1/1 (100)	-	1/10 (10.0)
***Mus*. *caroli***	-	-	-	-	-
***M*. *cervicolor***	-	-	-	-	-
***R*. *berdmorei***	0/1 (0)	-	-	-	0/1 (0)
***R*. *bukit***	0/2 (0)	-	-	0/1 (0)	0/3 (0)
***R*. *exulans***	-	13/45 (28.9)	0/1 (0)	0/1 (0)	13/47 (27.7)
***R*. *losea***	0/1 (0)	-	-	-	0/1 (0)
***R*. *norvegicus***	0/1 (0)	1/1 (100)	1/2 (50.0)	0/2 (0)	2/6 (33.3)
***R*. *rattus***	5/104 (4.8)	2/11 (18.2)	17/28 (60.7)	1/10 (10.0)	25/153 (16.3)
***R*. *sabanus***	0/6 (0)	-	-	0/2 (0)	0/8 (0)
***R*. *surifer***	0/10 (0)	-	-	0/1 (0)	0/11 (0)
**Total**	**5/287 (1.7)**	**16/62 (25.8)**	**20/35 (57.1)**	**6/170 (3.5)**	**47/554 (8.5)**

The lice were identified as *Polyplax* spp. (61.1%) and *Hoplopleura* spp. (38.9%), fleas were identified as *Xenopsylla cheopis*, and ticks were identified as *Haemaphysalis* spp. Mites collected from rats were more diverse than other types of ectoparasites. Three to five mites were selected from each pool and morphologically identified to genus (subgenus), and species if possible. They were all identified as trombiculid mites (Trombiculidae family, Trombiculinae subfamily). The most predominant genera were as follows: *Gahrliepia* (39.9%), *Leptotrombidium* (34.3%), *Ascoschoengastia* (14.6%), *Blankaartia* (5.4%), *Schoengastia* (4.0%), *Helenicula* (1.5%) and *Lorillatum* (0.3%). Two major genera collected from rats were further identified to subgenus. The results showed that within the genus *Gahrliepia*, subgenus *Walchia* was the most prevalent followed by *Schoengastiella*, and then *Gahrliepia*. Almost all mites in *Leptotrombidium* genus belonged to subgenus *Leptotrombidium*.

Among the tested ectoparasites, a high prevalence of *Bartonella* DNA was detected in lice (57.1%, 20/35) and fleas (25.8%, 16/62). High prevalence of *Bartonella* DNA was detected in 17/28 louse pools (60.7%) collected from *R*. *rattus* and 13/45 flea pools collected from *R*. *exulans*. Mites and ticks were mostly collected from *B*. *indica* and *R*. *rattus* and only 1.7% (5/287) of mite pools and 3.5% (6/170) of tick pools were positive for *Bartonella* DNA. Among *Bartonella*-positive trombiculid mites, 3 pools were *Leptotrombidium* genus, 1 pool was *Ascoschoengastia* genus, and the last pool did not have slide for morphological identification.

### Identification of *Bartonella* species in rodent hosts and their associated ectoparasites based on *glt*A sequence variations

**Fig 1:**
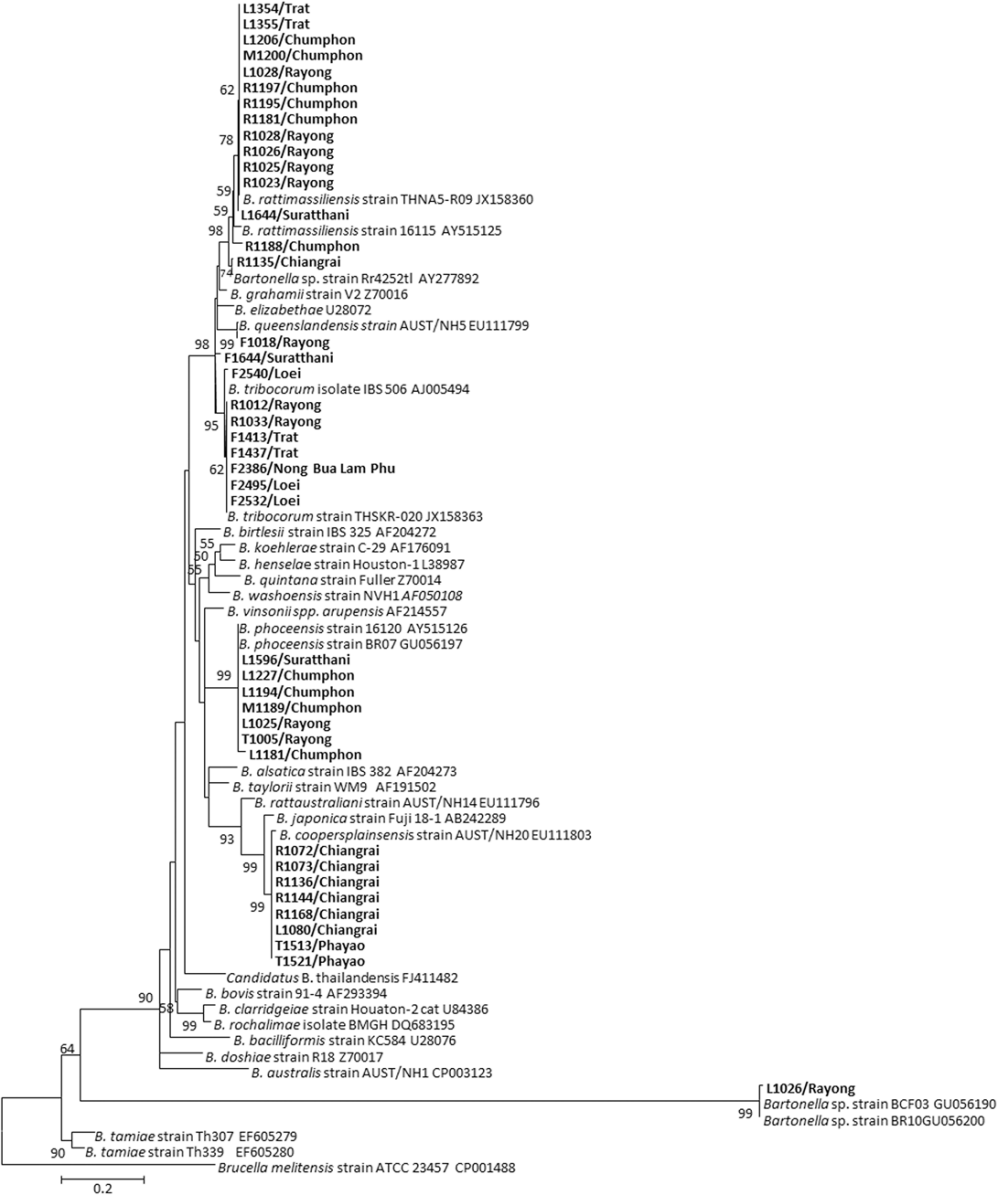
Phylogenetic relationship between *glt*A sequences of *Bartonella* species. *Bartonella* species detected from rodents and their associated ectoparasites; mite (M), flea (F),tick (T), and louse (L), along with reference sequences (GenBank accession numbers are noted after each sequence). Only bootstrap replicates of >50% are shown. The *Bartonella* species detected in this study are indicated in bold letters.

A total of 16 *Bartonella* isolates were successfully cultured from 26 individual rodent blood samples. *Bartonella* species detected from rats and ectoparasites based on sequences and phylogenetic analyses of 318 bp *glt*A amplicon are presented in Table 5 and Fig 1. Percent identity to the reference *Bartonella glt*A sequences of *Bartonella* species detected in this study are summarized in Table 6. Maximum-likelihood (ML) tree (Fig 1) shows the relationship between sequences generated from *Bartonella*-positive samples clustered into 8 different cladograms as described below. The majority of identified sequences (25/41 sequences) fell within *B*. *elizabethae* species complex. Within this *B*. *elizabethae* complex group (25 sequences), 15 *B*. *rattimassiliensis* were detected from 9 rats, 5 louse pools, and 1 mite pool sharing 96.5–100% identity with strain 16115 (AY515125) and strain THNA5-R09 (JX158360). Eight sequences of *B*. *tribocorum* were detected from 2 rats and 6 flea pools sharing 99.3–100% identity with strain IBS506 (AJ005494) and strain THSKR-020 (JX158363). One sequence of *B*. *queenslandensis* was detected from a flea pool sharing 99.6% identity with strain AUST/NH5 (EU111799). One sequence of undescribed *Bartonella* species within *B*. *elizabethae* complex was detected from a flea pool (#F1644) with a percent identity ranging from 96.8 to 97.1% to *B*. *tribocorum* strain IBS506 (AJ005494) and strain THSKR-020 (JX158363). The rest of the detected *Bartonella* sequences (16 sequences) were identified into eight *B*. *coopersplainsensis* which were detected from 5 rats, 2 tick pools, and 1 louse pool sharing 99.0–100% identity with strain AUST/NH20 (EU111803). Seven *B*. *phoceensis* were detected solely from ectoparasites: 5 in louse pools, 1 in a mite pool, and 1 in a tick pool sharing 98.4–100% identity with *B*. *phoceensis* strain 16120 (AY515126) and *B*. *phoceensis* strain BR07 (GU056197). Interestingly, one sequence obtained from *Bartonella*-positive louse pool (#L1026) was distantly related to the rest of the *Bartonella* species (66.3–68.2% identity) detected in this study, however, it was similar to some new strains of *Bartonella* species strain BCF03 (GU056190) and strain BR10 (GU056200) obtained from Taiwan with 99.3% identity (Table 6).

**Table 5:** *Bartonella* species identified in rodents and their associated ectoparasites based on *glt*A gene sequence similarities

*Bartonella* species (*glt*A)	Rodents	Mites	Fleas	Lice ^c^	Ticks	Total
***B*. *rattimassiliensis***	9	1^a^	-	5	-	**15 (36.6%)**
***B*. *tribocorum***	2	-	6	-	-	**8 (19.5%)**
***B*. *elizabethae***	-	-	-	-	-	**-**
***B*. *queenslandensis***	-	-	1	-	-	**1 (2.4%)**
**Other *Bartonella* of *B*. *elizabethae* species complex**	-	-	1	-	-	**1 (2.4%)**
***B*. *phoceensis***	-	1^b^		5	1	**7 (17.1%)**
***B*. *coopersplainsensis***	5	-	-	1	2	**8 (19.5%)**
**New *Bartonella* species (GU056190)**	-	-	-	1^d^	-	**1 (2.4%)**
**Total**	**16 (39.0%)**	**2 (4.9%)**	**8 (19.5%)**	**12 (29.3%)**	**3 (7.3%)**	**41 (100%)**

^a^ *L*. *deliense*.

^b^ Mite species was not available since there were only 3 mites collected from this host and no slide was made for species identification.

^**c**^ *Bartonella* DNA was equally detected in *Polyplax* and *Hoplopleura* lice.

^d^ *Polyplax* spp.

**Table 6:** Percent similarity of *glt*A sequence for *Bartonella* identification detected from rodents (R) and their associated ectoparasites; Mite (M), flea (F), tick (T), and louse (L).

	% Similarity											
Names	*B*. *elizabethae*	*B*. *rattimassiliensis strain 16115*	*B*. *rattimassiliensis strain THNA5-R09*	*B*. *queenslandensis strain AUST/NH5*	*B*. *tribocorum isolate IBS506*	*B*. *tribocorum strain THSKR-020*	*B*. *phoceensis strain 16120*	*B*. *phoceensis strain BR07*	*B*. *coopersplainsensis strain AUST/NH20*	*B*.*grahamii* strain V2	*Bartonella* species strain BCF03	*Bartonella* species strain BR10
F1644	95.9	93.4	94	95.6	**97.1**	96.8	88.4	88.4	85.9	96.5	67.2	67.2
R1135	94	**97.1**	**97.1**	93.1	94.7	94.3	87.8	87.8	85.3	95.3	67.2	67.2
R1188	92.8	**96.5**	**96.5**	92.2	92.8	92.5	87.8	87.8	85	94.3	67.2	67.2
Group 1^a^	93.1	97.5	**100**	92.2	93.4	93.1	87.2	87.2	84.1	94.3	66.6	66.6
L1644	93.4	97.8	**99.6**	92.5	93.7	93.4	87.5	87.5	84.4	94.7	66.3	66.3
F1018	93.7	92.2	92.5	**99.6**	94	93.7	87.5	87.5	83.4	95.3	67.9	67.9
F2540	94.7	92.2	92.8	94.3	**99.3**	99	88.1	88.1	85.6	95.3	67.2	67.2
Group 2^b^	94.3	92.5	93.1	93.4	99.6	**100**	89	89	85.9	95.6	67.2	67.2
Group 3^c^	88.1	87.2	87.2	87.8	88.7	89	**100**	**100**	88.7	90.3	67.6	67.6
L1181	87.5	86.2	86.2	87.2	87.8	88.1	**98.4**	**98.4**	87.8	89.4	67.6	67.6
Group 4^d^	85.6	85.6	85	84.4	86.9	86.6	89.7	89.7	**99**	87.5	66.6	66.6
L1026	66.6	65.4	66.3	67.6	67.2	66.9	67.2	67.2	66.3	68.2	**99.3**	**99.3**

^a^ Group 1 consists of sample no. R1023, R1025, R1026, R1028, R1181, R1195, R1197, L1028, M1200, L1206, L1354, and L1355.

^b^ Group 2 consists of sample no. R1012, R1033, F1413, F1437, F2386, F2495, and F2532.

^c^ Group 3 consists of sample no. T1005, L1025, M1189, L1194, L1227, and L1596.

^d^ Group 4 consists of sample no. R1072, R1073, R1136, R1144, R1168, L1080, T1513, and T1521.

The distribution of *Bartonella* species found in the North and northeast seems to be a region-specific distribution. Four samples of *B*. *tribocorum* were found in the Northeast and 8 samples of *B*. *coopersplainsensis* were detected in the North, even though one *B*. *rattimassiliensis* was also detected in the North (Fig 1 and Table 5). In contrast, *Bartonella* species found in samples collected from the East and the South are quite diverse consisting of six different species; *B*. *rattimassiliensis*, *B*. *queenslandensis*, *B*. *tribocorum*, *Bartonella* species within *B*. *elizabethae* species complex, and a presumably new strain of *Bartonella* species.

In this study, we found that the distribution of *Bartonella* species among rodents supports a host-specific pattern. Thus, five *B*. *coopersplainsensis* were solely detected from *B*. *indica*. Almost all *B*. *rattimassiliensis* (8/9) were detected from *R*. *rattus*, while two *B*. *tribocorum* were detected from *R*. *norvegicus* only. However, no specific pattern has been observed among ectoparasites. For example, two different *Bartonella* species (*B*. *rattimassiliensis* and *B*. *phoceensis*) were detected in mite pools (Table 5), 3 species (*B*. *tribocorum*, *B*. *queenslandensis* and other *Bartonella* of *B*. *elizabethae* species complex) were detected in flea pools (*Xenopsylla cheopis)*, 4 species (*B*. *rattimassiliensis*, *B*. *phoceensis*, *B*. *coopersplainsensis* and new *Bartonella* species (GU056190)) were detected in louse pools (*Polyplax* spp. and *Hoplopleura* spp.), and 2 species (*B*. *phoceensis* and *B*. *coopersplainsensis*) were detected in tick pools (*Haemaphysalis* spp.).

### Prevalence and distribution of *Bartonella* species detected in ectoparasites collected from *Bartonella*-positive and *Bartonella*-negative rats

The difference of *Bartonella* DNA prevalence in ectoparasites collected from *Bartonella*-positive and *Bartonella*-negative rats was investigated and presented in Table 7. Among *Bartonella*-positive rats, 20/103 (19.4%) ectoparasite pools were positive for *Bartonella* species. In contrast, only 27/309 (8.7%) ectoparasite pools were positive from *Bartonella*-negative rats. Of these *Bartonella*-positive ectoparasites, louse pools possessed the highest prevalence of 65% (13/20) and 46.7% (7/15) for positive and negative rats, respectively (Table 7). In general, *Bartonella* DNA prevalence in ectoparasites collected from positive rats (19.4%) were higher significantly (Chi-Square Tests, *P* = 0.003) comparing to ectoparasites from negative rats (8.7%).

**Table 7:** Comparison *of Bartonella* prevalence in ectoparasites collected from *Bartonella*-positive and *Bartonella*-negative rodents.

	Prevalence of *Bartonella* species in vectors among *Bartonella*-positive rodent			Prevalence of *Bartonella* species in vectors among *Bartonella*-negative rodents		
Ectoparasites	No. of positive	No. of infested rodent host	% positive	No. of positive	No. of infested rodent host	% positive
Mites	4	69	5.8	1	218	0.5
Fleas^a^	1	4	25	15	50	30
Ticks^a^	2	10	20	4	26	15.4
Lice	13	20	65	7	15	46.7
**Total**	**20**	**103**	**19.4** ^**b**^	**27**	**309**	**8.7** ^**b**^

^a^ There are 2 samples where *Bartonella* was detected in host (*R*. *rattus*), mite, and lice; 1–4 pools of ticks and fleas can be collected from one rodent.

^b^ *Bartonella* DNA prevalence in ectoparasites collected from positive rats (19.4%) were higher significantly (Chi-Square Tests, *P* = 0.003) comparing to ectoparasites from negative rats (8.7%).

## Discussion

Our study highlights the surveillance of *Bartonella* species among rodents and their associated ectoparasites (ticks, fleas, lice, and mites) in several regions across Thailand. The data demonstrated the high prevalence of *Bartonella* DNA in rats and their associated ectoparasites, especially in lice and fleas, as well as the finding of a diverse range of *Bartonella* species circulated among them. Previous studies have shown the high prevalence of *Bartonella* species among rats captured from Chiang Rai province (8.7%) [34] and from several regions across the country (41.5%) [35]. Although the prevalence in the latter study by Bai *et al*. was higher (41.5%, 137/330) than what we found (17.6%, 109/619), the proportion of rodent species are relatively the same except that *B*. *indica* was the highest number trapped in our study (45.1%, 279/619), while Bai *et al*. reported *R*. *rattus* as the most prevalent species investigated in their study (65.2%, 88/135). Given the higher rate of *Bartonella*-positive detected in *R*. *rattus* comparing to *B*. *indica* rat, the high *Bartonella* prevalence reported in Bai’s study can be explain by rodent species composition. Similarly, high prevalence (41.3%) of *Bartonella* was reported in rodents and shrews in Taiwan with the highest prevalence was found in *R*. *norvegicus* (52.7%) [36].

Although *Bartonella* DNA was detected in all types of ectoparasites (ticks, lice, fleas, and mites) collected from rats in this study, the prevalence varied substantially between ectoparasite types. The highest rate was found in lice and fleas that were in agreement with the study conducted in Taiwan [20]. Though *Bartonella* DNA was found in lower prevalence in ticks and mite pools in both studies, Kabeya *et al*. reported high prevalence in mites (82.9%) collected from rats in Thailand and all *Bartonella* species detected from mites were identified into *B*. *tamiae* based on their DNA sequences [15]. Although we do not believe that rat-associated lice can transmit pathogens to humans because of their high specificity to the host, for example, *Polyplax* and *Hoplopleura* lice are more likely specific to *Rattus* rats or to some other relative rat species [37, 38], the circulation of bartonellae via different ectoparasites, including lice, can influence the diversity of a rat-associated *Bartonella* community.

A diverse range of *Bartonella* species were isolated from whole blood of rats and shrews, including *B*. *rattimassiliensis*, *B*. *grahamii*, *B*. *elizabethae*, *B*. *tribocorum*, *B*. *coopersplainensis*, *B*. *phoceensis*, *B*. *queenslandensis*, and unknown genogroup [20, 32, 35, 36]. While only three of these species, *B*. *rattimassiliensis*, *B*. *tribocorum*, *B*. *coopersplainensis*, were isolated from rats in our study. *Bartonella* species found in rodent-associated ectoparasites were more diverse with seven different species found, including species isolated from their rat hosts. In this study, *glt*A gene sequence was used to identify *Bartonella* species, although ss*r*A gene was also sequenced and analyzed. We found that both genes were effective to identify *Bartonella* at the genus level; however, *glt*A gene sequence was found to be more suitable for species identification than *ssr*A gene sequence since the *glt*A sequence has expressed a higher range of variation comparing to the *ssrA*. Moreover, the availability of *glt*A reference sequences for *Bartonella* species in available databases is much higher than for *ssr*A genes that makes it a reliable and accurate tool for *Bartonella* species identification. Additionally, phylogenetic trees constructed from *Bartonella* culture isolates using both *ssr*A and *glt*A genes created quite similar phylogenetic tree topologies as shown in S1 Fig.

Kabeya *et al*. [15] reported the detection of *B*. *tamiae*, previously isolated from Thai febrile illness patients, in mites and tick pools collected from rats in Thailand. Two dominant mite genus most infected with *B*. *tamiae* were *Leptotrombidium* (66.7%) and *Schoengastia* (78.6%), therefore, the author suggested the role of mite as potential vector for *B*. *tamiae* transmission to human patients. In this study, *Bartonella* DNA was also detected from mites of *Leptotrombidium* and *Ascoschoengastia* genera, although *B*. *tamiae* was not found in our study. Our finding supports the role of trombiculid mite as a vector for *Bartonella* transmission. Because of the difficulty of mite species identification, the mite species in each pool was determined based on 3–5 mites selected from each pool and mounted on a glass slide as mentioned in materials and methods.

From our study, we found that *Bartonella* positive ectoparasites from *Bartonella*-positive rats was higher than in *Bartonella*-negative rats suggesting that this environment promotes the occurrence of horizontal transmission of *Bartonella* bacteria during the bite/feeding of vectors on rats. However, in order to prove infection route, transmission studies from an infected vector to naïve hosts/rats should be done in a controlled laboratory environment. Seasonal dynamics of *Bartonella* infection in natural populations of rats can also obscure a correlation between prevalence in rats and ectoparasites.

*Bandicota* and *Rattus* rats are the most common reservoir hosts for *Bartonella* infection in Southeast Asia which raises a question about the potentially important role of these rodent-borne agents as sources of febrile illness in human populations in Thailand. Detection of *Bartonella* DNA similar to *B*. *tamiae*, isolated from three febrile patients [23], and from mites collected from rodents in Thailand [15] imply a potential connection role for transmission of the disease to humans. Data from Kosoy et al. 2010, support this potential transmission route came from isolation of *Bartonella* species, previously identified from rodent hosts, from Thai patients’ blood and supported by analysis of history of the patients having an exposure to rats during the 2 weeks before the illness [35]. *Bartonella* species identified in these human cases included *B*. *elizabethae*, *B*. *rattimassiliensis*, and *B*. *tribocorum*, and the last two of these species were also detected in rodents, mites, fleas, and lice reported in this study.

## Disclaimer

Research was conducted in compliance with the Animal Welfare Act and other federal statutes and regulations relating to animals and experiments involving animals and adheres to principles stated in the Guide for the Care and Use of Laboratory Animals, NRC Publication, 2011 edition. The opinions or assertions contained herein are the private views of the author, and are not to be construed as official, or as reflecting true views of the Department of the Army or the Department of Defense.

## Supporting Information

S1 Fig Phylogenetic relationships between *Bartonella* species isolated from rodent blood and some reference species according to the *ssr*A and *glt*A phylogenies. The GenBank accession numbers are shown for each reference sequences. Trees constructed from *ssr*A and *glt*A genes were able to discriminate all samples into two major clusters (C1 and C2), although some different branching patterns of sequences in C1 group (R1012, R1033, R1135, and R1188) were noticed.
(TIF)

S1 File Additional data of ectoparasites collected from rodents and *Bartonella* DNA prevalence in the developmental stages of tick and flea. Detail information of ectoparasites collected from rodents in this study (Table A).*Bartonella* DNA detection in flea pools classified by gender (Table B) and in tick pools classified by stage and gender (Table C)
(DOCX)
