# Supplementary figures and images for: The Distribution and Diversity of Bartonella Species in Rodents and Their Ectoparasites across Thailand
Source: PLoS One. 2015 Oct 20;10(10):e0140856. doi: 10.1371/journal.pone.0140856 (PMC4617648; doi:10.1371/journal.pone.0140856)

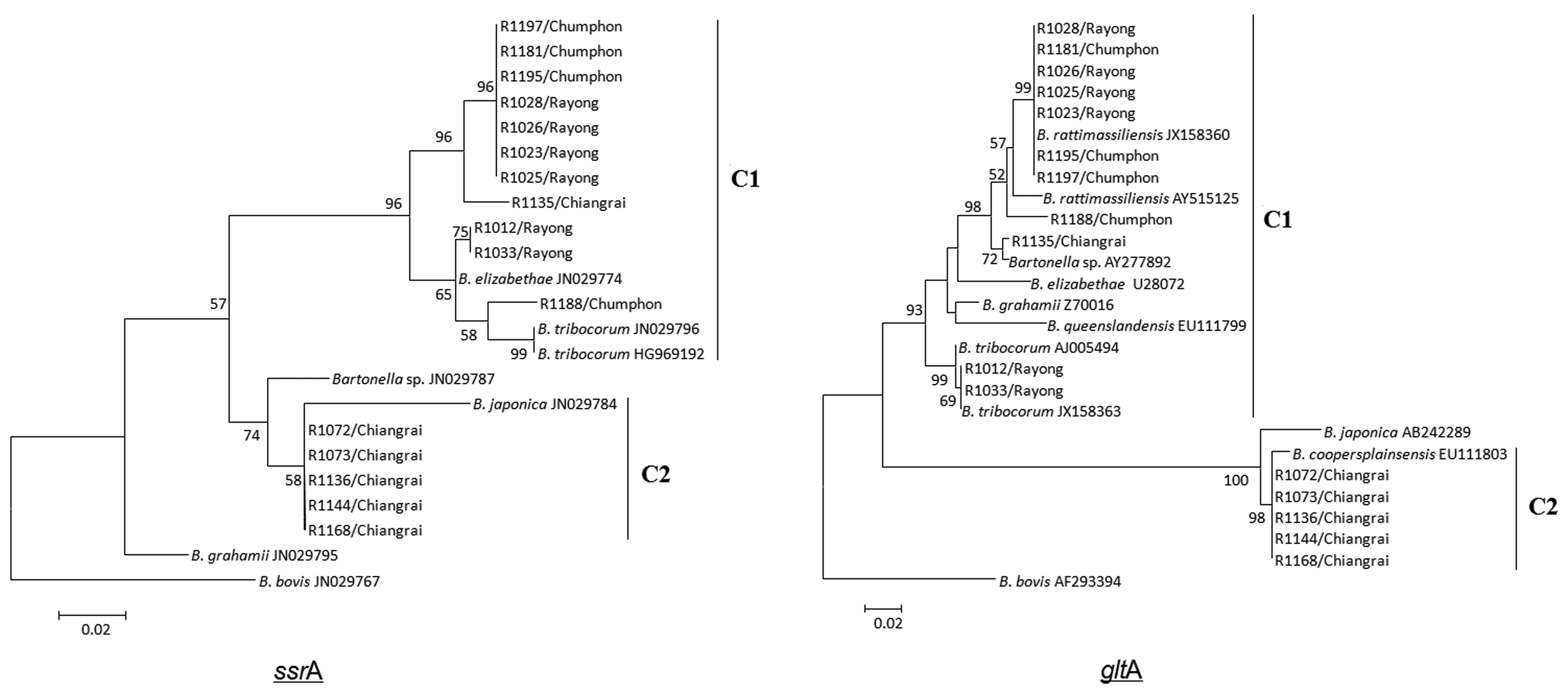

Supplement: S1 Fig — The GenBank accession numbers are shown for each reference sequences. Trees constructed from ssrA and gltA genes were able to discriminate all samples into two major clusters (C1 and C2), although some different branching patterns of sequences in C1 group (R1012, R1033, R1135, and R1188) were noticed. (TIF) [file pone.0140856.s001.tif]
