# Supplementary material for: The Distribution and Diversity of Bartonella Species in Rodents and Their Ectoparasites across Thailand
Source: PLoS One. 2015 Oct 20;10(10):e0140856. doi: 10.1371/journal.pone.0140856 (PMC4617648; doi:10.1371/journal.pone.0140856)
Supplement: S1 File — Detail information of ectoparasites collected from rodents in this study (Table A).Bartonella DNA detection in flea pools classified by gender (Table B) and in tick pools classified by stage and gender (Table C) (DOCX) [file pone.0140856.s002.docx]

**Table A. Information of ectoparasites collected from rodent in this study.**

| **Region** | **Provinces** | **Number of rodent collected** | **Number of rodents without ectoparasites** | **Number of rodents infested with ectoparasites** | **Number of rodent with > 2 types of ectoparasites** | **Rodent-associated ectoparasites** | **Number of rodent infested with pool(s) of ectoparasites** | | | |
| --- | --- | --- | --- | --- | --- | --- | --- | --- | --- | --- |
|  |  |  |  |  |  |  | **Total pool** | **1 pool** | **2 pools** | **> 4 pools** |
| **North** | Chiangrai | 138 | 79 | 59 | 0 | Mite | 57 | 57 |  |  |
|  |  |  |  |  |  | Fleas | 0 | 0 |  |  |
|  |  |  |  |  |  | Louse | 2 | 2 |  |  |
|  |  |  |  |  |  | Tick | 0 | 0 |  |  |
|  | Phayao | 61 | 32 | 29 | 10 (Chigger + Tick) | Mite | 15 | 15 |  |  |
|  |  |  |  |  | 2 (Flea+Tick) | Fleas | 2 | 2 |  |  |
|  |  |  |  |  |  | Louse | 0 | 0 |  |  |
|  |  |  |  |  |  | Tick | 157 | 2 | 2 | 20 |
| **Northeast** | Loei | 53 | 17 | 36 | 6 (Chigger + Tick) | Mite | 25 | 25 |  |  |
|  |  |  |  |  |  | Fleas | 11 | 11 |  |  |
|  |  |  |  |  |  | Louse | 0 | 0 |  |  |
|  |  |  |  |  |  | Tick | 7 | 5 | 1 |  |
|  | Nong Bua Lam Phu | 42 | 17 | 25 | 0 | Mite | 8 | 8 |  |  |
|  |  |  |  |  |  | Fleas | 26 | 10 | 8 |  |
|  |  |  |  |  |  | Louse | 1 | 1 |  |  |
|  |  |  |  |  |  | Tick | 0 | 0 |  |  |
| **East** | Rayong | 39 | 19 | 20 | 4 (Chigger + Lice) | Mite | 14 | 14 |  |  |
|  |  |  |  |  | 2 (Chigger + Tick) | Fleas | 2 | 2 |  |  |
|  |  |  |  |  | 2 (Tick + Lice + Chigger) | Louse | 8 | 8 |  |  |
|  |  |  |  |  |  | Tick | 6 | 6 |  |  |
|  | Trat | 101 | 64 | 37 | 0 | Mite | 15 | 15 |  |  |
|  |  |  |  |  |  | Fleas | 20 | 20 |  |  |
|  |  |  |  |  |  | Louse | 4 | 4 |  |  |
|  |  |  |  |  |  | Tick | 0 | 0 |  |  |
| **South** | Chumphon | 79 | 6 | 73 | 16 (Chigger + Lice) | Mite | 73 | 73 |  |  |
|  |  |  |  |  |  | Fleas | 0 | 0 |  |  |
|  |  |  |  |  |  | Louse | 16 | 16 |  |  |
|  |  |  |  |  |  | Tick | 0 | 0 |  |  |
|  | Surat Thani | 106 | 25 | 81 | 2 (Chigger + Lice) | Mite | 80 | 80 |  |  |
|  |  |  |  |  | 1 (Chigger + Lice + Flea) | Fleas | 1 | 1 |  |  |
|  |  |  |  |  |  | Louse | 4 | 4 |  |  |
|  |  |  |  |  |  | Tick | 0 | 0 |  |  |
| Grand Total  (# pool) |  | 619 | 259 | 360 |  |  | 554 | 381 | 22 | 151 |

**Table B. *Bartonella* DNA detection in flea pools classified by gender**.

| **Province** | **No. of *Bartonella* DNA positive/total collected (% positive)** | | |
| --- | --- | --- | --- |
|  | **Flea gender** | | **Grand Total** |
|  | **female** | **male** |  |
| Loei | 3/9 (33.3) | 0/2 (0) | 3/11 (27.3) |
| Nong Bua Lam Phu | 1/14 (7.1) | 1/12 (8.3) | 2/26 (7.7) |
| Phayao | 0/2 (0) | - | 0/2 (0) |
| Rayong | 1/2 (50.0) | - | 1/2 (50.0) |
| Surat Thani | 1/1 (100.0) | - | 1/1 (100.0) |
| Trat | 9/20 (45.0) | - | 9/20 (45.0) |
| **Grand Total** | 15/48 (31.3) | 1/14 (7.1) | 16/62 (25.8) |

**Table C**. ***Bartonella* DNA detection in tick pools classified by stage and gender**.

| **Province** | **No. of *Bartonella* DNA positive/total collected (% positive)** | | | | |
| --- | --- | --- | --- | --- | --- |
|  | **Tick stage/gender** | | | | **Grand Total** |
|  | **female** | **male** | **nymph** | **larva** |  |
| Loei | 0/2 (0) | - | - | 0/5 (0) | 0/7 (0) |
| Phayao | 5/68 (7.4) | 0/44 (0) | 0/10 (0) | 0/35 (0) | 5/157 (3.2) |
| Rayong | 1/1 (100.0) | 0/5 (0) | - | - | 1/6 (16.7) |
| **Grand Total** | 6/71 (8.5) | 0/49 (0) | 0/10 (0) | 0/40 (0) | 6/170 (3.5) |

Note: No tick was collected from rodents in Nong Bua Lam Phu, Surat Thani, and Trat.
